# Magnetoencephalography biomarkers for assessing myelin content and neuronal function in acute optic neuritis

**DOI:** 10.1093/braincomms/fcag218

**Published:** 2026-06-10

**Authors:** Ysoline Beigneux, Christophe Gitton, Abdul Rauf Anwar, Jean-Didier Lemarechal, Mariem Hamzaoui, Michel Paques, Catherine Vignal, Isabelle Audo, Benedetta Bodini, Bruno Stankoff, Letizia Leocani, Catherine Lubetzki, Nathalie George, Céline Louapre

**Affiliations:** Department of Neurology, CIC Neurosciences, Sorbonne Université, Paris Brain Institute—ICM, Assistance Publique Hôpitaux de Paris, Inserm, CNRS, Hôpital de la Pitié Salpêtrière, Paris 75013, France; Sorbonne Université, Institut du Cerveau—Paris Brain Institute—ICM, Inserm, CNRS, APHP, Hôpital de la Pitié Salpêtrière, CENIR, Centre MEG-EEG, Paris 75013, France; Sorbonne Université, Institut du Cerveau—Paris Brain Institute—ICM, Inserm, CNRS, APHP, Hôpital de la Pitié Salpêtrière, CENIR, Centre MEG-EEG, Paris 75013, France; Institut de Neurosciences de la Timone, Aix Marseille Université, Marseille 13005, France; Sorbonne Université, Paris Brain Institute—ICM, Inserm, CNRS, Paris 75013, France; Sorbonne Université, INSERM, CNRS, UMR_S 968, Institut de la Vision, Paris 75012, France; Centre Hospitalier National d'Ophtalmologie des Quinze-Vingts, INSERM-DHOS Clinical Investigation Center 1423, Paris 75012, France; Centre Hospitalier National d'Ophtalmologie des Quinze-Vingts, Centre de Référence Maladies Rares REFERET and INSERM-DGOS CIC 1423, Paris 75012, France; Department of Neuro-Ophthalmology, Foundation Adolphe de Rothschild Hospital, Paris 75019, France; Sorbonne Université, INSERM, CNRS, Institut de la Vision, Paris 75012, France; Centre Hospitalier National d'Ophtalmologie des Quinze-Vingts, Unité d’électrophysiologie Centre de Référence Maladies Rares REFERET and INSERM-DGOS CIC 1423, Paris 75012, France; Department of Neurology, CIC Neurosciences, Sorbonne Université, Paris Brain Institute—ICM, Assistance Publique Hôpitaux de Paris, Inserm, CNRS, Hôpital de la Pitié Salpêtrière, Paris 75013, France; Department of Neurology, CIC Neurosciences, Sorbonne Université, Paris Brain Institute—ICM, Assistance Publique Hôpitaux de Paris, Inserm, CNRS, Hôpital de la Pitié Salpêtrière, Paris 75013, France; Experimental Neurophysiology Unit, Institute of Experimental Neurology-INSPE, IRCCS San Raffaele Scientific Institute, Milan 20132, Italy; Department of Neurology, CIC Neurosciences, Sorbonne Université, Paris Brain Institute—ICM, Assistance Publique Hôpitaux de Paris, Inserm, CNRS, Hôpital de la Pitié Salpêtrière, Paris 75013, France; Sorbonne Université, Institut du Cerveau—Paris Brain Institute—ICM, Inserm, CNRS, APHP, Hôpital de la Pitié Salpêtrière, CENIR, Centre MEG-EEG, Paris 75013, France; Department of Neurology, CIC Neurosciences, Sorbonne Université, Paris Brain Institute—ICM, Assistance Publique Hôpitaux de Paris, Inserm, CNRS, Hôpital de la Pitié Salpêtrière, Paris 75013, France

**Keywords:** MEG, VEP, OCT, demyelination, neurodegeneration

## Abstract

The visual pathway is an important model system for remyelination and neuroprotection trials in multiple sclerosis, due to its accessibility and the availability of validated methods including visual evoked potential and optical coherence tomography. However, visual evoked potentials are sometimes undetectable and demonstrate limited reliability after acute optic neuritis. This study aims to investigate novel magnetoencephalography markers for assessing myelin content and neuronal dysfunction in the early phase of optic neuritis and describes their inter-run reproducibility (‘over a single visit’) and association with short-term visual outcomes. Patients with unilateral acute optic neuritis were recruited and underwent ophthalmological assessments, brain MRI and magnetoencephalography. Magnetoencephalography data were acquired during visual stimulation with an alternating checkerboard pattern. We used source localization to reconstruct brain activity in the primary visual cortex (V1) and analysed it in the temporal and frequency domains. In the temporal domain, we focused on M100 latency—the magnetic counterpart of P100 latency. In the frequency domain, we assessed the spectral richness of the steady-state evoked field response by harmonic count, which reflects the diversity of frequency components present in the brain signal. Thirty-two patients were included at a median of 54 days [interquartile range = (37.5–78)] post-symptom onset of optic neuritis. Among patients with optic neuritis, visual evoked field recordings were detectable in 77% of cases, compared with 66% for visual evoked potential recordings. M100 latency demonstrated an excellent inter-run reproducibility for both fellow and affected eyes [intra-class correlation coefficient (ICC) >0.8, mean absolute inter-run difference of 2.99 ± 6.53 and 3.76 ± 7.53 ms, respectively]. By comparison, the reproducibility of P100 latency was good for fellow eye (ICC = 0.7, mean absolute inter-run difference of 3.9 ± 6.2 ms) but moderate for affected eye (ICC = 0.6, mean absolute inter-run difference of 9.1 ± 21.8 ms). In the frequency domain, the harmonic count correlated strongly with ganglion cell layer volume (*r* = 0.68, *P* = 0.0001), likely reflecting functional consequences of neuronal loss. Measures reflecting demyelination (P100 and M100 latencies) correlated with measures of neuronal damage (ganglion cell layer volume and harmonic count) from both conventional and magnetoencephalography assessments. Visual impairment was associated with neuronal damage (parameter estimates: *β* = 0.49, *P* = 0.017 for ganglion cell layer volume, *β* = 0.57, *P* = 0.003 for harmonic count) but not with demyelination measures. Our results highlight magnetoencephalography as a reproducible and comprehensive tool to study both myelin content and neuronal dysfunction shortly after optic neuritis and suggest that, at this early stage, neuronal damage is already the main driver of visual outcome.

## Teaser

Beigneux *et al*. showed that magnetoencephalography can assess both optic nerve myelin content and neuronal dysfunction after optic neuritis, a common manifestation of multiple sclerosis. Spectral richness, reflected by harmonic content, captures neuronal dysfunction and is associated with visual disability shortly after acute optic neuritis.

## Graphical Abstract

**Graphical Abstract:**
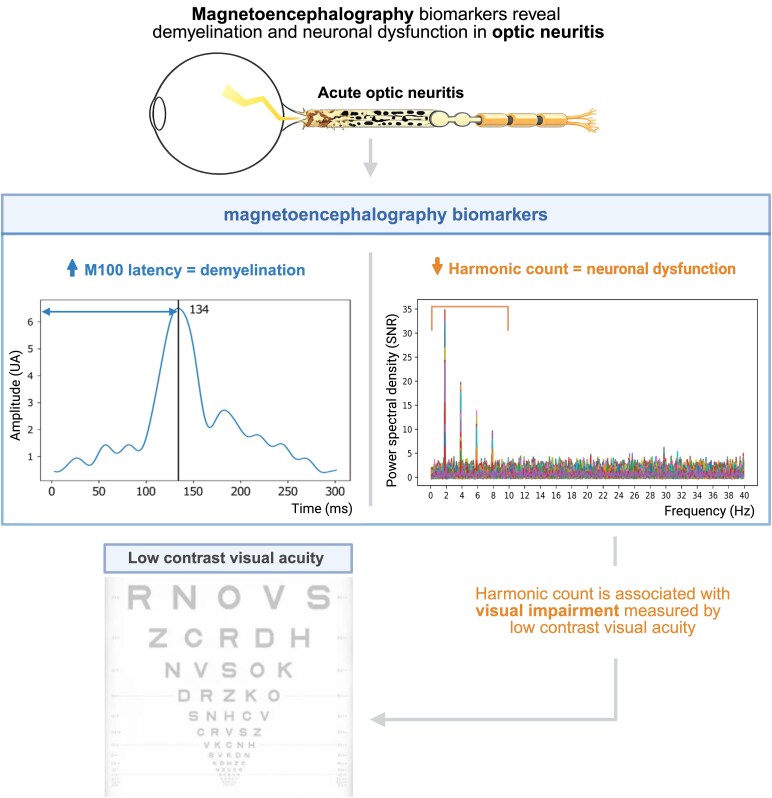

## Introduction

In multiple sclerosis , significant research efforts are focused on developing therapies that promote remyelination and prevent neuronal loss.^1^ Nonetheless, designing remyelination clinical trials and selecting appropriate outcomes remain a significant challenge.^2^ Visual outcome measures, notably visual evoked potentials (VEPs) in acute optic neuritis (ON) or in chronic optic neuropathy, have gained prominence in remyelination trials, as they directly reflect the dynamics and the functional character of demyelination and remyelination of the optic nerve through changes in P100 latency.^3^ Additionally, optical coherence tomography (OCT) complements VEP by measuring the thickness of the ganglion cell layer (GCL), providing insights into neuronal loss within the optic nerve.^4^

Two remyelination trials have shown positive results in patients with chronic optic neuropathy, in the form of a 1.7-ms shortening in P100 latency with clemastine,^5^ and 4.75-ms shortening with bexarotene in a subgroup of patients who had delayed baseline P100 latency and no clinical ON in the preceding 5 years.^6^

No therapeutic remyelination trial conducted in the acute phase has yet demonstrated clear or statistically significant effects on disease or symptom progression, although some studies, such as the RENEW trial of opicinumab, showed trends towards improved conduction recovery.^7,8^ Yet, experimental data suggest a ‘window of opportunity’ for remyelination in the acute phase of lesion formation, before the occurrence of neuronal loss. In contrast, in chronic lesions, the persistent neuroinflammatory environment may be detrimental to remyelination.^9^ A significant challenge in pinpointing this ‘window of opportunity’ in patients with multiple sclerosis lies in the low reliability and reproducibility of P100 measurement during the acute phase, due to dispersed waveforms. Additionally, VEPs are sometimes not recordable due to conduction block associated with inflammation.^10,11^

Other methods of electrophysiological brain activity recording could allow tackling this challenge. Magnetoencephalography (MEG) directly measures neuronal ensemble activity by recording magnetic fields associated with postsynaptic currents at the scalp level. It offers excellent temporal resolution on the order of milliseconds. Additionally, source localization methods combined with co-registration with structural MRI enhance spatial resolution, particularly because magnetic fields, unlike electric potentials in EEG, are not distorted by their propagation throughout different brain structures, including skull and scalp.^12,13^ While visual evoked fields (VEFs) recorded by MEG in response to visual periodical stimuli have been documented in healthy individuals with good reproducibility,^14,15^ no study has yet been published in patients with ON.

Beyond time-domain analysis, MEG—as well as EEG—data can also be analysed in the frequency domain, notably through steady-state visual evoked potential and field (SSVEP/F). SSVEP/F reflect the oscillatory brain response induced by repetitive visual stimulation; it shows a prominent peak at the frequency of the stimulation (fundamental frequency) and at multiples of this frequency (harmonics). SSVEP/F provide an excellent signal-to-noise ratio and offer objective (i.e. at a predetermined frequency) and quantifiable markers of visual brain responses without the need for an explicit task.^16^ This approach has been used to study both low-level visual processes (e.g. in ophthalmology and basic vision research^17^) and for assessing visual recognition. Recent study emphasized the importance of studying harmonics, which are an integral component of brain responses.^18^

The objective of this study was to explore the potential of MEG for revealing alterations in visual responses during the early phase after acute ON. We investigated source-localized magnetic evoked responses and activities in the frequency domain from V1 region. We hypothesize that the latency of magnetic evoked responses could serve as a reproducible marker of myelin content, while a reduction in spectral richness may reflect neuronal dysfunction, part of which could be reversible. This measure could provide complementary information to OCT, which primarily assesses irreversible neuronal degeneration. Together, these novel biomarkers may help disentangle the contributions of demyelination and neuronal injury to visual impairment in ON and clarify their respective roles in early disease processes.

## Materials and methods

### Participants

Thirty-two patients with multiple sclerosis or clinically isolated syndrome (CIS), between 18 and 60 years old, diagnosed with acute ON were included in the ON-STIM trial (NCT 04042363), a therapeutic trial evaluating the potential effect of electric optic nerve stimulation on remyelination, conducted between February 2020 and August 2023 at Pitié-Salpêtrière Hospital, Paris. Patients were included from 15 days to 3 months between the last relapse medical treatment of ON (corticosteroids and plasma exchanges if necessary) and inclusion. The results presented in this study are based on pre-treatment data acquired during the screening visit.

Inclusion and exclusion criteria are detailed in Supplementary Methods.

Twelve healthy subjects, between 18 and 60 years old, with similar age and sex distribution of patients, were included as controls.

Written informed consent was obtained from all participants, and the study was approved by the Ethics Committee of CPP Ile de France 8.

### Ophthalmological assessments

- Standard visual tests were performed, including high-contrast visual acuity (HCVA), low-contrast visual acuity (LCVA), Lanthony Test and Humphrey visual field testing (24-2 Swedish Interactive Thresholding Algorithm). OCT scans were performed using a Spectralis® spectral domain OCT device. The volume (in mm^3^) of the GCL was estimated. Pattern reversal VEPs were recorded monocularly at the Hospital of Ophthalmology of Quinze-Vingts. VEP traces were reviewed by L.L.

The delay between the VEP and MEG recordings was <1 week. Full details of ophthalmological assessments are presented in Supplementary Methods.

### MRI acquisition

All subjects underwent whole-brain structural MRI (3-T Prisma with a 32-channel head coil; Siemens). The acquired MRI sequences and the processing for source localization are detailed in the Supplementary Methods. The Human Connectome Project (HCP) multimodal parcellation scheme (HCP-MMP1.0) was used to define V1 in each subject.

### Magnetoencephalography acquisition

#### Stimuli

The MEG protocol was based on the same stimuli and task as the VEP assessment. The stimuli consisted of black-and-white checkerboard pattern reversal with an alternating rate of 2 Hz and three inner-square sizes (15′, 30′ and 60′). They were generated and displayed using Psychtoolbox 3.0.18 for MATLAB 2015b. The average luminance of the entire pattern was 13.77 cd/m^2^, and the Michelson contrast between the black and white squares of the checkerboard was 88.56%.

#### Magnetoencephalography data acquisition and experimental protocol

The data were acquired at the MEG Centre of the Research Neuroimaging Centre core facility of Paris Brain Institute. Data were acquired continuously at 1000 Hz with a low-pass anti-aliasing filter of 130 Hz. in a magnetically shielded room with a whole-head 306-channel Elekta Neuromag® Triux MEG system, which comprises 2 orthogonal planar gradiometers and 1 magnetometer at each of 102 positions.

The MEG protocol was similar to the VEP assessment and consisted of four monocular stimulation runs (two for each eye). The order of eye stimulation was counterbalanced across participants. Each stimulation run comprised three blocks of 60 s duration, each corresponding to one square size (15′, 30′ and 60′), presented in a random order. Two patients were excluded due to claustrophobia (*n* = 1) and excessive drowsiness (*n* = 1) during MEG acquisition.

### Magnetoencephalography data analyses

#### Pre-processing

Pre-processing is detailed in Supplementary Methods. Further data analysis was performed with MNE Python (version 23.0).

#### Visual evoked field source analysis

VEFs were obtained at the MEG channel level by averaging the epochs of 500 ms after stimulus onset, for each participant. The epochs were baseline-corrected using the 150 ms interval preceding each stimulus onset. Only the data from the 60′ size checkerboard were used for VEF computation, as this size was usually used in clinical trials. The VEFs were then projected into source space using the dynamic statistical parameter mapping (dSPM) inverse operator and taking the norm of the sources. Source localization method is detailed in Supplementary Methods.

#### Frequency domain steady-state visual evoked field source analysis

MEG data were analysed in the frequency domain to obtain the SSVEF in response to the alternating checkerboards. All sizes of squares (15′, 30′ and 60′) were included for this analysis. The checkerboard reversal period was 500 ms, generating a steady-state brain response at the fundamental frequency of 2 Hz and its harmonics, which were multiples of the fundamental frequency (4, 6, 8, … Hz).

To increase the signal-to-noise ratio, for each participant and each eye, we first averaged the six blocks of 60 s each from both runs. These data were then cropped into five overlapping epochs of 20 s duration. These epochs were projected into source space using dSPM inverse operator. The multi-taper method was then applied to compute the power spectrum density (PSD) of the source space data, resulting in a PSD estimation for each one of the 8196 sources contained in the surface-based cortical source space, ranging from 0 to 40 Hz, with a resolution of 0.05 Hz. The PSDs of the five overlapping epochs were averaged together for each participant.

The various MEG processing steps in both the temporal and frequency domains are summarized in Supplementary Fig. 1.

#### Measurements of visual evoked field and steady-state visual evoked field parameters

Data from the two acquisition runs and from both left and right V1 areas were averaged, except in the analysis of reproducibility, for both VEF and SSVEF source measurements. We extracted metrics of interest in V1 area from the VEF and SSVEF source analyses described above.

- M100 latency (equivalent of P100 in VEP): This was defined as the peak of the mean signal from all sources in left and right V1 (80 ± 17 sources). For each hemisphere, eye, subject and run, the VEF extracted from V1 was displayed, highlighting local maxima between 0 and 300 ms. Two independent evaluators (Y.B. and C.Lo.), blinded to the subjects and their characteristics, identified the peak corresponding to the M100 wave. The same procedure was performed for the P100 wave in VEP, with recordings from O1 and O2 analysed separately. In addition, we examined the spatial variability of M100 peak latency across the sources in left and right V1. This analysis is detailed in Supplementary Methods.
- Harmonic count: The PSDs obtained from SSVEF analysis were averaged across all sources in V1, and significant power peaks at the fundamental and harmonic frequencies were defined using *Z*-scores. These *Z*-scores were obtained at each frequency by computing the amplitude at the considered frequency bin minus the mean amplitude of the surrounding frequency bins (10 on either side), skipping the closest neighbour bin on each side of the considered frequency bin and dividing this value by the standard deviation of the amplitude of the 20 surrounding bins.^19^ Harmonic count was defined as the number of harmonics in which the amplitude was significantly above noise, using a *Z*-score >4.

### Statistical analyses

The statistical analyses were performed using Python 3.8 and R 3.6.3.

#### Reproducibility of the P100/M100 latencies and of harmonic counts

Test–retest (aka. inter-run) and inter-rater reproducibility of the P100 and M100 latencies were assessed using the intra-class correlation coefficient (ICC).^20^ We also computed the inter-run reproducibility of the harmonic count using ICC. ICC values were interpreted as follows: <0.5 indicates poor reproducibility, 0.5–0.75 moderate reproducibility, 0.75–0.9 good reproducibility and >0.9 excellent reproducibility.^21^

Mean absolute differences between runs and between raters were calculated. Test–retest variability (TRV, in ms) was calculated as the standard deviation of the distribution of per cent change values of P100 and M100 latencies between runs and raters.

#### Differences between affected and fellow eyes for harmonic count

Differences in metrics between affected and fellow eyes were analysed using two-sided paired *t*-test across subjects, with *P* < 0.05 as the significance level. Normally distributed data were checked for M100 latency, P100 latency, GCL volume and harmonic count.

#### Correlation between Magnetoencephalography and ophthalmological measures

Association between numerical variables was analysed using Pearson’s correlation (package SciPy).

#### Multiple linear regression models of visual impairment outcome

First, we performed a principal component analysis on LCVA, HCVA and the Lanthony test scores and took the first component of this principal component analysis (PCA) as a composite index of visual impairment.

Second, we built two multiple linear regression models to explore the association between the visual index and measures of demyelination and neuronal damage, using the function lm() of lme4 package:

- Conventional Ophthalmological Model: Visual index ∼ macular GCL + P100 latency.
- MEG Model: Visual index ∼ harmonic count + M100 latency.

The independent predictor variables were centred on zero and standardized using *Z*-scoring for comparisons across different scales and measures.

Residuals, q–q plots, scale-location plots and residual versus leverage plots were visually checked to ensure appropriate model fits. To prevent issues related to multicollinearity, the variance inflation factors were checked for all independent variables, confirming that values were less than two.

For each model, the significance of the predictors was determined using *t*-tests (with significance threshold at *P* < 0.05).

We also evaluated how well each model explained the variance of the visual index, as assessed by, first, adjusted *R*-squared values, and second, Akaike Information Criterion (AIC), which allows model comparison (see Supplementary Methods for details).

## Results

### Demographic, clinical, electrophysiological and imaging characteristics

Thirty-two patients were included in the study at a median of 54 days [interquartile range (IQR) = (37.5–78)] following the onset of ON symptoms. Demographic, clinical, electrophysiological and imaging characteristics are detailed in Table 1.

**Table 1:** Demographic, clinical, electrophysiological and imaging characteristics

Subject-level characteristics	Patients with ON (*n* = 32)		Healthy subjects (*n* = 12)
Age, years	33 ± 7.1		33 ± 7.5
Gender (female)	21 (66%)		8 (67%)
CIS/multiple sclerosis	8/24 (75%)		
EDSS, median (IQR)	1.25 (1.0–2.0)		
Corticosteroids for ON treatment	32 (100%)		
Days from ON onset to inclusion, median (IQR)	54 (37.5–78)		
ON length (mm) (*n* = 30)	17.5 ± 6.5		
Visual parameters	Affected eye (*n* = 32)	Fellow eye (*n* = 32)	
Clinical visual assessments			
HCVA letters (number)	78.8 ± 15.7	83.3 ± 12.9	
2.5% LCVA (number)	14.2 ± 14.3	24.2 ± 15.2	
Lanthony test (error number)	10.35 ± 4.55	7.3 ± 5.3	
Visual field			
Mean deviation (dB)	−5.0 ± 5.9	−3.3 ± 4.8	
Visual field index (%)	89 ± 19.0	93 ± 14.9	
Foveal threshold (dB)	30.6 ± 8.8	33.7 ± 7.3	
Retinal OCT			
pRNFL thickness (µm)	88.9 ± 17.9	93.6 ± 15.8	
GCL volume (mm^3^)	0.89 ± 0.17	0.99 ± 0.18	
Electrophysiology			
P100 latency in VEP (ms)	131 ± 13.7 (*n* = 28)	105 ± 8.3 (*n* = 31)	
M100 latency in VEF (ms)	130.1 ± 17.4 (*n* = 29)	107.4 ± 14.8 (*n* = 30)	102 ± 6.0

Results are expressed as number (%) or mean ± standard deviation, unless specified otherwise.

EDSS, Expanded Disability Status Scale; pRNFL, peripapillary retinal nerve fibre layer.

### Examples of visual evoked potential and visual evoked field

**Figure 1:**
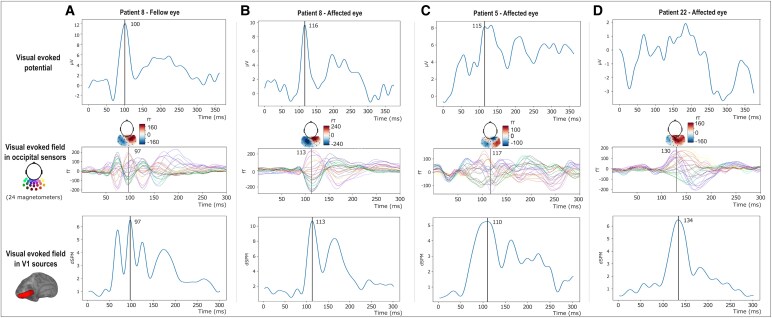
**VEP (first row), magnetic VEF on occipital magnetometer sensors (second row) and in V1 sources (third row) for example affected and fellow eyes.** The vertical black line and the number next to it in each plot indicate P100 or M100 latencies. Right hemisphere responses are shown on this figure. (**A**) In Patient 8, fellow eye VEP and VEF demonstrated classical components (N75, P100 and N135) in all modalities, while affected eye VEF (**B**) showed a delay in P100/M100 and the disappearance of N75 peak. (**C**) In Patient 5, the affected eye VEP and VEF illustrated a dispersed response in all modalities, with bifid peak in VEP, variable waves across occipital sensors and a better-defined peak in V1 sources. (**D**) In Patient 22, there was no identifiable evoked response in the VEP of the affected eye, while the M100 could be identified in VEF both at occipital-sensor and V1 source levels. fT, femtotesla.

Figure 1 illustrates examples of VEP and VEF on occipital magnetometer sensors and VEF extracted from V1 sources, in response to visual stimulation of affected and fellow eyes. Different modifications of patterns were observed after ON. Source reconstruction restricted to the V1 area yielded a more focal magnetic evoked response than at the sensor level, facilitating precise identification of the M100 latency (Fig. 1C). The magnetic N75 component faded in 4 out of 32 patients, suggesting that ON may lead to complex electrophysiological changes beyond a simple P100/M100 component delay (Fig. 1B). Figure 1D illustrates a challenging P100 latency individualization in VEP, while M100 latency appeared more detectable, particularly at the source level.

Source estimates over time on the cortical surface following stimulation of the fellow eye are presented in Supplementary Video 1 (lateral view) and Supplementary Video 2 (medial view).

### Reproducibility of M100 latency in V1 sources

We carefully inspected VEF time courses on occipital sensors and in V1 sources in all patients. Because we intended to measure M100 latency independently of head position within the MEG helmet for further longitudinal studies, we decided to measure M100 latency reproducibility based on the source signals extracted from V1.

In patients, the VEF recordings from 7 out of 30 (23.3%) affected eyes were excluded due to undetectable responses in at least one of the acquisition runs. No fellow eye VEF recording was excluded (30/30 analysed), and all eyes from healthy subjects were analysed. None of the 30 patients with undetectable VEF responses exhibited a detectable response on VEP.

**Figure 2:**
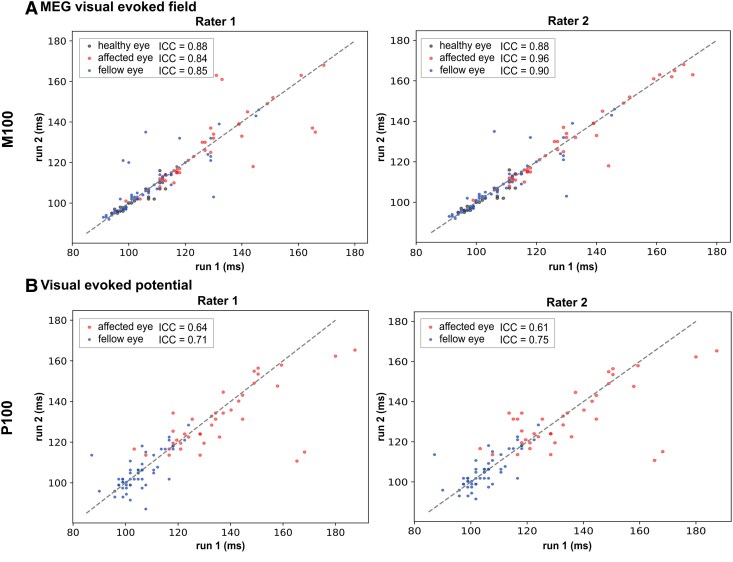
**Inter-run reproducibility plots for (A) M100 latency (in ms) measured in VEF V1 sources and (B) P100 latency (in ms) measured in VEP.** Each data point represents a measurement from both eyes and both hemispheres for each patient. Red: Affected eyes (*N* = 23 for VEF and *N* = 21 for VEP); blue: fellow eyes (*N* = 30 for VEF and 29 for VEP); grey: healthy subjects (*N* = 24).

Table 2 presents the results of M100 latency reproducibility analysis. In healthy subjects, M100 latency inter-run ICC was good (ICC = 0.88 for both raters), and inter-rater reproducibility was perfect (ICC = 1). For patients’ fellow eye, M100 latency reproducibility was good to excellent (inter-run ICC 0.85 and 0.90), with a mean absolute inter-run difference of 2.99 ± 6.53 ms. For patients’ affected eye, inter-run ICC was also good to excellent (0.84 and 0.96), with a mean absolute inter-run difference of 3.76 ± 7.53 ms (Fig. 2A). Inter-rater M100 latency reproducibility was excellent for both affected and fellow eyes (ICC = 0.99 and 0.95, respectively).

**Table 2:** VEF M100 latency reproducibility between runs and between raters

	Rater	Eye	ICC (95% CI)	Absolute difference (ms)	TRV (ms)
Inter-run reproducibility	1	Healthy subjects	0.88 (0.75–0.95)	1.91 ± 2.58	3.86
		FE	0.85 (0.76–0.91)	3.33 ± 7.10	9.82
		AE	0.84 (0.73–0.91)	4.95 ± 10.21	14.02
	2	Healthy subjects	0.88 (0.75–0.95)	1.91 ± 2.58	3.86
		FE	0.90 (0.83–0.94)	2.65 ± 5.95	8.17
		AE	0.96 (0.94–0.98)	2.57 ± 4.85	6.75
Inter-rater reproducibility		Healthy subjects	1 (1–1)	0.0 ± 0	0
		FE	0.99 (0.98–0.99)	0.35 ± 2.78	3.87
		AE	0.95 (0.93–0.97)	2.06 ± 7.77	11.08

Fellow eye: *n* = 30 (one chart excluded for excessive drowsiness and another for claustrophobia). Affected eye: *n* = 23 (due to undetectable VEF response in at least one run).

CI, confidence interval; FE, fellow eye; AE, affected eye.

We also performed reproducibility analysis of P100 latency obtained from VEP for the sake of comparison with M100 latency marker. VEPs issued from the 60′ size checkerboard stimulation were excluded in 11 out of 32 (34%) affected eyes due to undetectable responses in at least one of the acquired runs. Three out of 32 fellow eye VEP recordings were excluded (only 1 acquired run in 2 patients and undetectable response in 1 patient with previous ON).

Supplementary Table 1 presents the results of P100 latency reproducibility analysis. For patients’ fellow eye, P100 latency reproducibility was moderate to good (inter-run ICC = 0.71 and 0.75), with a mean absolute inter-run difference of 3.9 ± 6.2 ms. For patients’ affected eye, inter-run ICC was moderate (0.64 and 0.61), with a mean absolute inter-run difference of 9.1 ± 21.8 ms (Fig. 2B). Inter-rater P100 latency reproducibility was excellent for both affected and fellow eyes (ICC = 0.98 and 0.99, respectively).

Supplementary Figure 2A and B shows M100 variability across the individual V1 sources in the fellow and affected eyes of an example patient. M100 latency variance across the 80 ± 17 V1 sources was significantly higher for the affected than for the fellow eyes (33 ± 1.96 versus 23 ± 1.70 ms^2^, respectively, *P* < 0.001) (Supplementary Fig. 2C).

The correlation between M100 variance across V1 sources and M100 latency was not significant for either fellow or affected eyes (*r*  *=* 0.25, *P* = 0.20 and *r* = −0.35, *P* = 0.065). Thus, M100 dispersion and M100 latency appeared as at least partly independent metrics.

### Harmonic count in V1 steady-state visual evoked field responses

**Figure 3:**
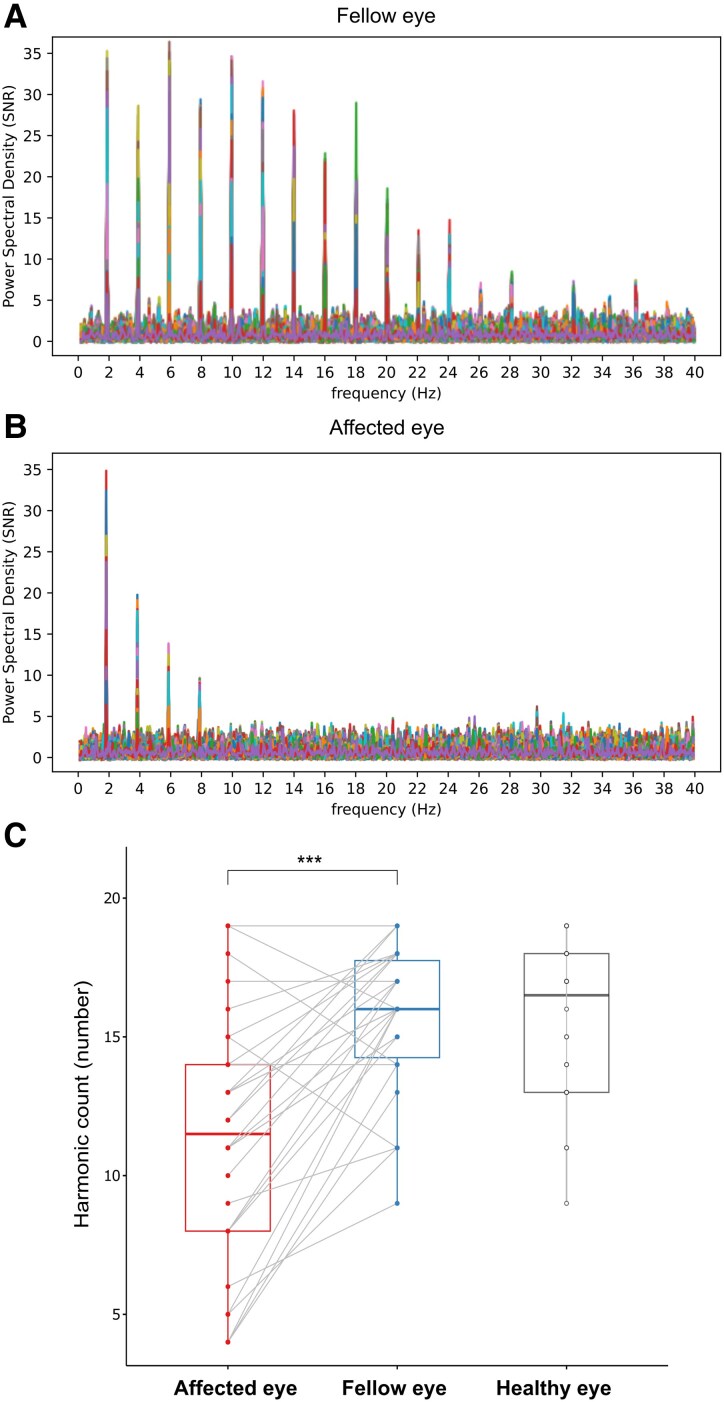
**Analysis in the frequency domain and harmonic count.** Example of spectral representation for all the sources in V1 (different colours) after stimulation of a fellow eye (**A**) and stimulation of an affected eye (**B**). Two Hz corresponds to the fundamental frequency, and multiples of fundamental frequency correspond to harmonics. Harmonic count is defined by the number of harmonics in which the amplitude was significantly above noise using a *Z*-score >4. (**C**) Box plot of harmonic count calculated in affected (*N* = 29), fellow (*N* = 29) and healthy eyes (*N* = 24). Each data point represents a measurement from each eye for each patient. Both eyes are displayed for the healthy subject group. Harmonic count was significantly lower for affected eyes compared with fellow eyes (***P < 0.001 by paired *t*-test).

In order to capture the spectral content of the signal recorded during the visual stimulation, we analysed the SSVEF from V1 sources in the frequency domain. We excluded one patient with no light perception leading to the absence of spectral response. Therefore, PSD analysis and harmonic count were conducted in 29 patients and 12 healthy subjects. Figure 3 shows examples of spectral representations exhibiting the fundamental 2 Hz response to visual stimulation and its harmonics for a fellow eye (Fig. 3A) and an affected eye (Fig. 3B).

The harmonic count was significantly lower in affected eyes [median = 10, IQR = (6–12)] than fellow eyes [median = 14, IQR = (12–16), *P* < 0.001] (Fig. 3C).

Inter-run reproducibility of harmonic count was deemed good for affected eyes (ICC = 0.78) and excellent for fellow eyes (ICC = 0.90).

### Associations between magnetoencephalography metrics in V1, electrophysiology and imaging parameters

**Figure 4:**
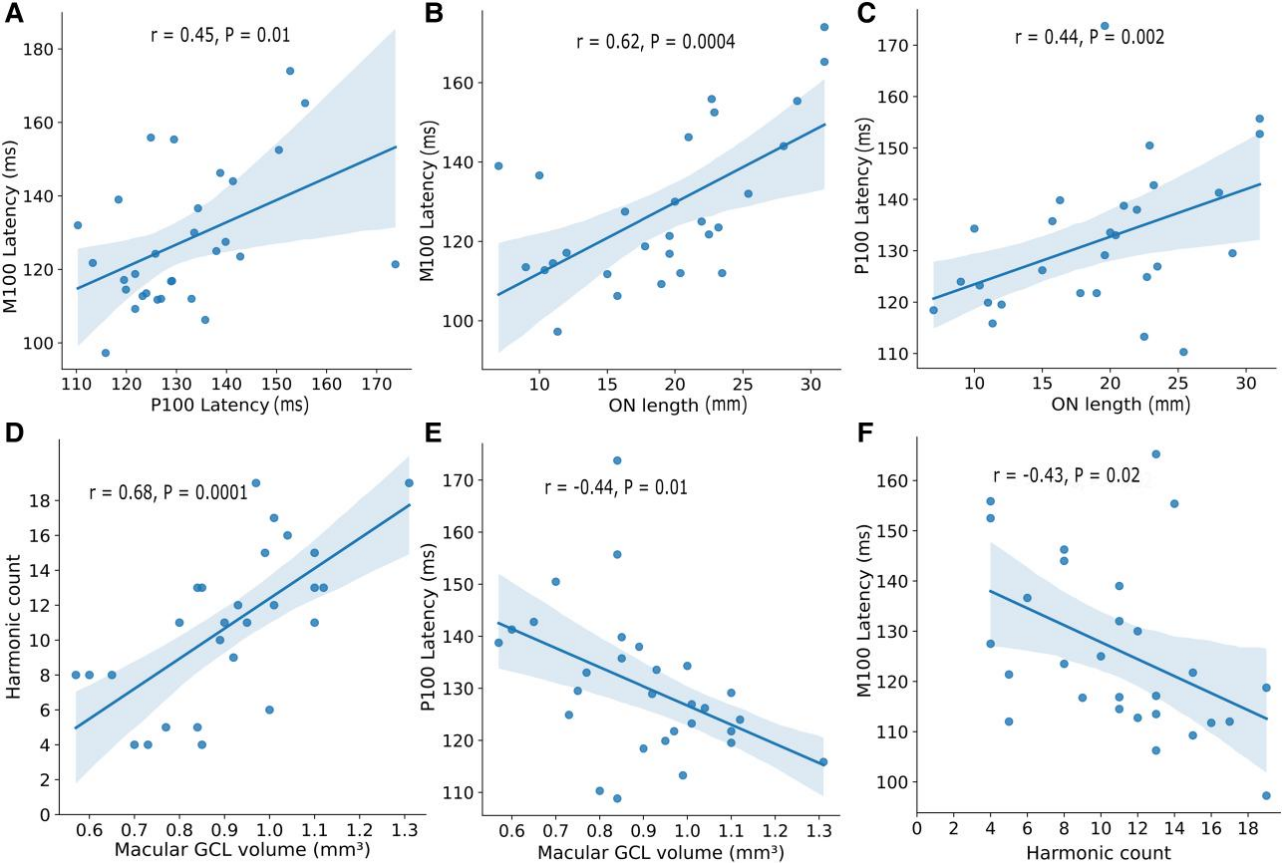
**Correlation between MEG, MRI and ophthalmological measures for affected eyes.** (**A**) P100 latency and M100 latency correlated for the affected eyes. Significant correlations were found between ON length and M100 latency (**B**) and P100 latency (**C**). (**D**) Harmonic count correlated strongly with macular GCL volume. (**E**) P100 latency correlated with macular GCL volume. (**F**) M100 latency correlated with harmonic count. Each data point represents a measurement from each affected eye for each patient. *N* = 29 except for **B** and **C**, *N* = 28. Pearson correlation coefficient *r* and *P*-values (*t*-test) are presented in each scatter plot.

M100 latency correlated with P100 latency for both fellow and affected eyes [*r* = 0.52, *P* = 0.002 and *r* = 0.45, *P* = 0.01 (Fig. 4A), respectively]. Moreover, both M100 latency and P100 latency correlated with ON length [*r*  *=* 0.62, *P* = 0.0004 (Fig. 4B) and *r* = 0.44, *P* = 0.002 (Fig. 4C), respectively].

The number of harmonics of the SSVEF in V1 correlated with GCL volume in affected eyes (*r*  *=* 0.68, *P* = 0.0001) (Fig. 4D) but not in fellow eyes (*r* = 0.20, *P* = 0.31).

### Correlation between demyelination and neuronal damage

We assessed the correlation between demyelination and neuronal damage based on the classical ophthalmological markers and on the new MEG-based markers. First, P100 latency correlated with macular GCL volume (*r* = −0.44, *P* = 0.01) (Fig. 4E). Second, similarly, M100 latency correlated with harmonic count (*r*  *=* −0.43, *P* = 0.02) (Fig. 4F). This suggests an association between demyelination and neuronal damage following acute phase of ON, as revealed by both types of markers.

### Relative contribution of demyelination and neuronal damage to visual impairment after acute optic neuritis

In order to better capture the heterogeneity of visual impairment among patients and to mitigate the floor effect of LCVA scores in patients with severe ON, we created a composite score called the visual index, defined as the first component of PCA of LCVA, HCVA and the Lanthony test scores. This first component accounted for 71% of the variance in these measures (with a loading of 0.64 for LCVA score, 0.53 for HCVA score and −0.56 for Lanthony test score). We defined the visual impairment index as the score projected on this first component. Lower visual index corresponded to more marked visual impairment.

Table 3 shows the results of two multiple linear regression model analyses exploring the association between the visual impairment index and (i) conventional ophthalmological parameters (‘Ophthalmological Model’) and (ii) MEG parameters (‘MEG Model’).

**Table 3:** Comparative analysis of ophthalmological and MEG models for determining visual impairment outcomes

	Estimate	Standard error	*t*-value	*P*-value
Ophthalmological model Adjusted *R*^2^ = 0.34, *F*-statistic = 7.7, *P*-value = 0.003, AIC = 70.2				
Intercept	4.4 × 10^−17^	0.16	0.00	1.00
P100 latency (ms)	−0.16	0.19	−0.87	0.39
GCL volume (mm^3^)	0.49	0.19	2 .57	0.017*

MEG model				
Adjusted *R*^2^ = 0.43, *F*-statistic = 11.0, *P*-value = 0.0004, AIC = 66.1				
Intercept	1.27 × 10^−16^	0.16	0.00	1.00
M100 latency (ms)	−0.10	0.17	−0.56	0.39
Harmonic count	0.57	0.18	3.24	**0**.003**

Multiple linear analysis for ophthalmological model and MEG model. Independent variables were centred. All variables were standardized using *Z*-score. Dependent variable is the visual index defined as first component of PCA performed on LCVA, HCVA and Lanthony test. Statistically significant *p*-values are reported in bold. *: *p* < 0.05; **: *p* < 0.005

In both models, the metrics reflecting neuronal damage (respectively, GCL volume and harmonic count), but not the metrics reflecting demyelination (P100 and M100 latencies), were significantly associated with visual impairment (parameter estimates: *β* = 0.49, *P* = 0.017 for GCL volume; *β* = 0.57, *P* = 0.003 for harmonic count).

The ‘MEG Model’ predicted visual impairment to marginally greater extent than the ‘Ophthalmological Model’ did, in terms of both adjusted *R*^2^ (43 versus 34%, respectively) and AIC values (66 versus 70, respectively) (Table 3).

## Discussion

This study, integrating MEG, MRI and ophthalmological evaluations, aimed to investigate alterations in visual responses during the early phase following an acute ON. The results showed that M100 latency measured at V1 source level is associated with demyelination, correlating with both P100 latency and ON length and demonstrating good reproducibility compared to P100 latency. Moreover, the analysis of SSVEF spectral content identified harmonic count as a marker of neuronal damage, as measured by GCL volume. Metrics reflecting neuronal damage, that is, macular GCL volume and harmonic count, were significantly associated with visual impairment, unlike measures of demyelination.

### Importance of reliable markers for exploring myelin dynamics in the early phase of optic neuritis

VEP latency is considered the gold standard marker of myelin dynamics during ON, reflecting changes in myelin content, as recently validated in animal models.^22^ Nonetheless, using VEP in the acute phase of ON is challenging leading to the exclusion of patients with undetectable VEP from remyelination trials (37.5% in simvastatin trial^23^ and 18% in opicinumab trial^7^). In our study, 34% of patients were excluded from repeatability analyses due to the absence of detectable P100 in at least one run, whereas a smaller proportion of 23.3% were excluded on the basis of M100 detectability in V1 sources, as responses could be obtained even in some patients with severe ON.

Moreover, previous studies showed that reproducibility of P100 latency is robust for subjects without optic nerve pathology but exhibits diminished reliability after ON.^11^ Indeed, Narayanan *et al.*’s^24^ study on full-field VEP) reported very good or good test–retest reliability for healthy or fellow eyes but a moderate reproducibility for eyes with chronic ON. To our knowledge, no reproducibility study has been conducted during the weeks following an ON. In our investigation, reproducibility for affected eyes was moderate, with a mean P100 latency absolute difference between the two runs of 9.1 ms. Interestingly, M100 latency inter-run reproducibility was markedly higher than that of VEP (mean absolute latency difference 3.8 ms). This higher reproducibility could be explained by the specific extraction of the evoked response in V1 allowed by source reconstruction with MRI co-registration. Indeed, VEF recorded over different occipital sensors (capturing activity from both V1 and higher-order visual areas) sometimes exhibits substantial latency differences. Specific analysis of V1 sources enabled us to obtain sharper evoked responses, facilitating the identification of the M100 latency and improving its reproducibility. Moreover, this extraction method based on anatomical landmarks with source reconstruction might be particularly valuable for longitudinal studies and could be useful to measure a potential surrogate for optic nerve myelin content in clinical trials.

That said, a remaining limitation of MEG is that it requires good patient compliance, as the subject must remain motionless, awake and should not be claustrophobic. In this respect, the advent of new types of sensors, which function at room temperature and do not require cooling by liquid helium (aka. optically pumped magnetometers) constitutes an important technological breakthrough.^25-27^

### Functional and structural biomarkers of neuronal impairment

Neuronal loss after ON can be quantified structurally using GCL volume measurements by OCT. However, GCL volume reflects irreversible neuronal loss and cannot capture reversible neuronal dysfunction. In contrast, functional electrophysiological measures may detect potentially reversible forms of neuronal impairment, notably associated with mitochondrial energy dysfunction.^28^ VEP amplitude has been shown to reflect neuronal dysfunction, but this measure demonstrates poor reproducibility, severely restricting its clinical utility.^11^ Therefore, there is a critical need for robust functional biomarkers to monitor neuronal dysfunction.

Previous studies have highlighted that frequency domain analysis with SSVEP/F stands out as a robust and objective method for scrutinizing cortical visual responses.^16^ In our study, we found that the harmonic count derived from SSVEF analysis in the frequency domain was markedly lower in affected eyes compared to fellow eyes and controls. This marker was reproducible and quantifiable even in severe cases of ON. Interestingly, for the affected eyes, harmonic count strongly correlated with GCL volume and was a major determinant of visual impairment, suggesting that this measure reflects neuronal damage in the optic nerve.

Harmonic generation requires synchronous activation of large neuronal populations. The presence of multiple frequencies in the response, which are not present in the stimulus, suggests that non-linear neural mechanisms contribute to the visual system response, reflecting the richness of neuronal ensemble response.^16^ The non-linear properties of the visual system may arise from the sequential activation of different dipoles from the retina to the visual cortex. Recent experiments employing artificial neural networks have successfully replicated the non-linear characteristics of the visual system.^29,30^ When neuronal dysfunction occurs, the number of retinal ganglion cells capable of transmitting synchronized signals to the visual cortex decreases. This neuronal depletion directly impacts the system's capacity to generate complex oscillatory responses: fewer functional axons result in reduced signal strength, leading to an impoverished harmonic spectrum with fewer detectable frequency components.

Due to its reproducibility and high signal to noise ratio, SSVEP/F harmonic count could be valuable in longitudinal studies to follow the temporality of neuronal dysfunction, which may be partly reversible. Further studies will also be needed to determine its prognosis value for visual recovery after ON.

### Association between demyelination, neuronal damage and visual impairment in the early phase of optic neuritis

In our cohort, we found a significant correlation between markers of demyelination and neuronal dysfunction using both MEG and ophthalmological metrics. Yet, although demyelination and neuronal damage were correlated, visual impairment was significantly predicted by the metrics of neuronal damage (harmonic count and GCL volume) but not by the metrics of demyelination (M100 and P100 latencies).

Previous studies have shown that GCL thickness predicts long-term visual disability after ON, including impaired colour vision^31^ and LCVA.^4,31,32^ Our findings extend these observations by demonstrating that this association is evident very early after ON and is linked, at least in part, to neuronal damage. Whether remyelination may prevent further neuronal damage or even restore neuronal function will require long-term follow-up of this cohort.

### Limitations

Our results are based on a cross-sectional pilot study, which included a limited number of patients and healthy control group. This study analysed inclusion data, which are part of a remyelination trial testing the efficacy of electrical stimulation to improve optic nerve remyelination. Consequently, the longitudinal analysis of results is restricted until the remyelination trial is complete.

Participants were recruited several weeks after ON onset (median time 54 days), in accordance with the ON-STIM trial inclusion criteria. Since neuronal degeneration occurs early, within the weeks following an episode, this recruitment window limits our ability to capture early structural changes and the dynamics of remyelination. Future studies including participants earlier in the course of acute ON will be essential to better understand the interplay between demyelination, remyelination and neurodegeneration. Moreover, a complementary study assessing test–retest reproducibility across separate visits would be necessary to estimate between-visit variability and to inform power calculations if MEG-derived measures are to be used as clinical trial endpoints. Despite a higher detection rate for M100 (77% of patients included) compared with P100 (66%), the most severe patients remain excluded from the analyses, which may limit the generalizability of the findings.

Although MEG is an expensive technique with limited availability, its use in remyelination trials appears promising. Importantly, lighter and less expensive devices are currently being developed,^33^ which could open new avenues for better characterizing the visual pathway in the context of acute or chronic ON.

## Conclusion

Using MEG coupled with ophthalmological and electrophysiological evaluations, our study underscores the complexity of characterizing the visual pathway in the context of acute ON and highlights the early impact of neuronal dysfunction on visual disability following ON. MEG, with its excellent temporal resolution and source reconstruction capabilities, provides a powerful tool for studying neuronal activity and neuronal transmission dysfunction, paving the way for improved therapeutic strategies and better clinical outcomes for affected patients in the early stages of ON.

## Supplementary Material

fcag218_Supplementary_Data

## Data Availability

Data not provided in the article because of space limitations may be shared (anonymized) at the request of any qualified investigator for purposes of replicating procedures and results. The code used for data analysis in this study is available in a public repository at: https://github.com/y-bgx/optic-neuritis-meg-paper.
