# Supplementary material for: Magnetoencephalography biomarkers for assessing myelin content and neuronal function in acute optic neuritis
Source: Brain Commun. 2026 Jun 10;8(3):fcag218. doi: 10.1093/braincomms/fcag218 (PMC13289812; doi:10.1093/braincomms/fcag218)
Supplement: fcag218_Supplementary_Data [file fcag218_supplementary_data.zip › Supplementary Materials.pdf]

Supplementary Materials

Supplementary Method

1. Participants

Inclusion criteria for patients were: age from 18 to 60 years old; MS (according to 2017 McDonald criteria[1]) with a duration of less than 10 years or CIS with dissemination in space on MRI; acute ON, optimally treated for the relapse (with corticosteroids and plasma exchanges if necessary); delay from 15 days to 3 months between last relapse medical treatment and inclusion.

Patients with atypical ON (papillitis, initial optic atrophy) or neuromyelitis optica were excluded. AQP4 testing was performed if the clinical or radiological presentation was suggestive of neuromyelitis optica spectrum disorder according to the established criteria.

MOGAD testing was performed in cases of severe ON, longitudinal optic nerve involvement, perineural optic sheath enhancement, or the presence of optic disc edema.

Inclusion criteria for healthy subjects were: no history of neurological or ophthalmological diseases and corrected visual acuity ≥ 0.8 in both eyes.

2. Visual assessment

Standard visual tests were performed, including:

- Best corrected high-contrast visual acuity (HCVA) using Early Treatment Diabetic Retinopathy Study (ETDRS) charts.
- Low contrast visual acuity (LCVA) using Sloan 2.5%, quantifying the number of letters correctly identified (0-60).
- Color perception using the 15-Hue Desaturated Test, calculating the number of errors.
- Humphrey visual field-testing, measuring the foveal threshold, the visual field index, and the mean deviation.

3. Optical coherence tomography

OCT scans were performed using a Spectralis® spectral domain OCT (SD-OCT) device (Heyex 7.0.8.0 Heidelberg Engineering, Germany) under standard conditions and with the eye-tracking modality without pupillary dilation. The peripapillary retinal nerve fiber layer (pRNFL) thickness (µm) was measured using a 12º diameter ring scan automatically centred on the optic nerve head (ART 30, HR A- Scans per B-Scan). Macular layer thicknesses were evaluated using a 20×15 degrees horizontal raster scan centred on the fovea (ART ≥9; 19 lines, 512 A-Scans per B-Scans, HR). Horizontal and vertical line scans were included. Intra-retinal layer segmentation was performed semi-automatically using the Spectralis segmentation algorithm (version 6.0c) and a 6 mm ring area grid, with manual correction of obvious errors by an orthoptist blind to the conditions. We estimated the thickness (in µm) of the retinal nerve fiber layer (RNFL) and the volume (in mm^3) of the inner nuclear layer (INL), outer plexiform layer (OPL), outer nuclear layer (ONL), photoreceptors (PRL) and ganglion cell layer (GCL). All SD-OCT scans met OSCAR-IB criteria.

4. Visual evoked potential

A MonPackONE setup with a CRT screen for stimulation (Metrovision, Perenchies, France) was employed following the recommendations of International Society for Electrophysiology in Vision (ISCEV).[2] Participants, wearing their optimal optic correction, were seated 100 cm away from the CRT screen, displaying a 2 Hz alternating black-and-white checkerboard with three different inner square sizes: 60', 30' and 15'. Stimulus screen luminance and contrast were kept constant throughout the study. The two recording electrodes were placed on the right and left occipital scalp (i.e. O1 and O2), with the reference electrode at Fz and the ground electrode on the earlobe. A minimum of 120 stimulus presentations of 500 ms each was averaged to obtain the visual evoked response allowing the measurement of P100 latency. At least 2 runs were recorded for each condition to evaluate reproducibility. Two independent evaluators (YB and CL), blinded to the subjects and their characteristics, identified the peak corresponding to the P100 wave for the right and left occipital recordings and for both runs independently.

5. MRI acquisition and processing for source localization

The MRI protocol included 3-dimensional T1-weighted magnetization-prepared rapid gradient-echo (3D-T1 MP2RAGE, RT/TE 5000/2.98 ms, inversion time 700 ms, resolution $1.0 \times 1.0 \times 1.0$ mm^3).

Optic nerve lesion length was measured on axial DIR FLAIR sequences centred on the optic nerves. (3D-DIR-FLAIR with fat saturation, RT/TE 7500/352 ms, inversion time 3000 ms, resolution 1.25 × 1.25 × 1.25 mm^3).
Segmentation of total grey matter, cerebral cortex, total white matter, and cerebrospinal fluid was performed on 3D-T1 MP2RAGE scans using Freesurfer 7.[3,4]
The Human Connectome Project (HCP) multimodal parcellation scheme (HCP-MMP1.0) was used to define anatomically the primary visual area V1 used for source reconstruction and analysis in MEG in each subject.

6. MEG data acquisition and experimental protocol

Four head position indicator (HPI) coils were used to monitor head position within the MEG helmet. Vertical and horizontal electro-oculograms (EOG) were recorded using disposable bipolar electrodes placed above and below the right eye and on the left and right outer canthi, respectively. Two additional disposable electrodes were placed over the subject's chest for measuring electrocardiogram (ECG). The 3D locations of the 4 HPI coils, head points across the scalp, and three anatomical fiducials (the nasion and left and right pre-auricular points) were recorded using a 3D digitizer (Fastrak Polhemus Inc.) before the subject was comfortably installed in the magnetically shielded room of the MEG system. The head position was determined at the beginning of each recording run. The delay between the trigger signal and the projection of stimuli (9 ms) was confirmed using a photodiode on the screen and was corrected during data processing. Eye gaze position and blinking were additionally monitored by an EyeLink 1000 eye-tracker at 1000 Hz. Participants were instructed to fixate a point at the center of the checkerboard.

7. MEG preprocessing

For each participant, to suppress external sources of noise, we first excluded artefactual noisy channels (presenting electronic “jumps”) by visual inspection and we preprocessed the raw MEG data using MaxFilter software (Maxfilter 2.2,[5] correlation limit of 0.98), with reconstruction of the excluded channels. Further preprocessing was performed with MNE Python (version 23.0). Eye-blink and cardiac artefacts were corrected using independent component analysis (ICA). High-pass filtering (> 1 Hz) was applied to compute ICA and we removed one independent component for eye-blink and one for heart artefacts, respectively.

8. MEG forward model for source localization

A surface-based cortical source space was derived from the segmented MRI of each individual (average spacing between equivalent current dipoles 9.9 mm, 8196 sources for the whole brain, not including the cerebellum). A loose orientation constraint of 0.2 was applied, favouring currents normal to the cortical surface by reducing the variance of the transverse source components. A single-layer boundary-element model (BEM) was determined from the inner skull surface and used as a head conductor model in the forward model computation (electrical conductivity = 0.3 S/m).

For the VEF analysis, we used the 150 ms before each checkerboard reversal onset as the baseline period for noise covariance matrix computation, in order to maximize the early evoked response component in the inverse model computation for VEF.

For the SSVEF analysis, resting state data were used to compute the noise covariance matrix. Resting state data was recorded during a 5-minutes run at the start of the recording session. During this run, participants fixated a cross in the centre of the screen.

9. Analysis of M100 latency variance across V1 sources

To examine the variability of M100 peak latency among the sources within left and right V1, we defined **M100 latency variance** as:

$$Variance = \frac{1}{n}\sum_{i=1}^{n}(x_i - \bar{x})^2 \quad (1)$$

where n is the number of sources in V1 area, x is the peak latency for each source i, and $\bar{x}$ is the mean M100 latency computed previously.

10. Akaike Information Criterion

AIC evaluates the trade-off between the model goodness of fit and its complexity. A lower AIC value indicates a model with a better fit, taking into account penalization for the number of predictors to mitigate overfitting.

Difference in AIC (ΔAIC) was interpreted [6] as follows:

• A ΔAIC of 0–2 suggests substantial evidence for difference between two models,

• A ΔAIC of 4–7 indicates considerable evidence for difference between two models,

• A ΔAIC greater than 10 represents very strong evidence for difference between two models.

References

1. Thompson AJ, Banwell BL, Barkhof F, et al. Diagnosis of multiple sclerosis: 2017 revisions of the McDonald criteria. *The Lancet Neurology*. 2018;17(2):162-173. doi:10.1016/S1474-4422(17)30470-2
2. Odom JV, Bach M, Brigell M, et al. ISCEV standard for clinical visual evoked potentials: (2016 update). *Doc Ophthalmol*. 2016;133(1):1-9. doi:10.1007/s10633-016-9553-y
3. Fischl B, Sereno MI, Tootell RB, Dale AM. High-resolution intersubject averaging and a coordinate system for the cortical surface. *Hum Brain Mapp*. 1999;8(4):272-284. doi:10.1002/(sici)1097-0193(1999)8:4<272::aid-hbm10>3.0.co;2-4
4. Dale AM, Fischl B, Sereno MI. Cortical surface-based analysis. I. Segmentation and surface reconstruction. *Neuroimage*. 1999;9(2):179-194. doi:10.1006/nimg.1998.0395
5. Taulu S, Simola J. Spatiotemporal signal space separation method for rejecting nearby interference in MEG measurements. *Phys Med Biol*. 2006;51(7):1759-1768. doi:10.1088/0031-9155/51/7/008
6. Burnham K, Anderson D. *Model Selection and Multimodel Inference : A Practical Information-Theoretic Approach*. Springer Verlag; 2002.

Supplementary Results

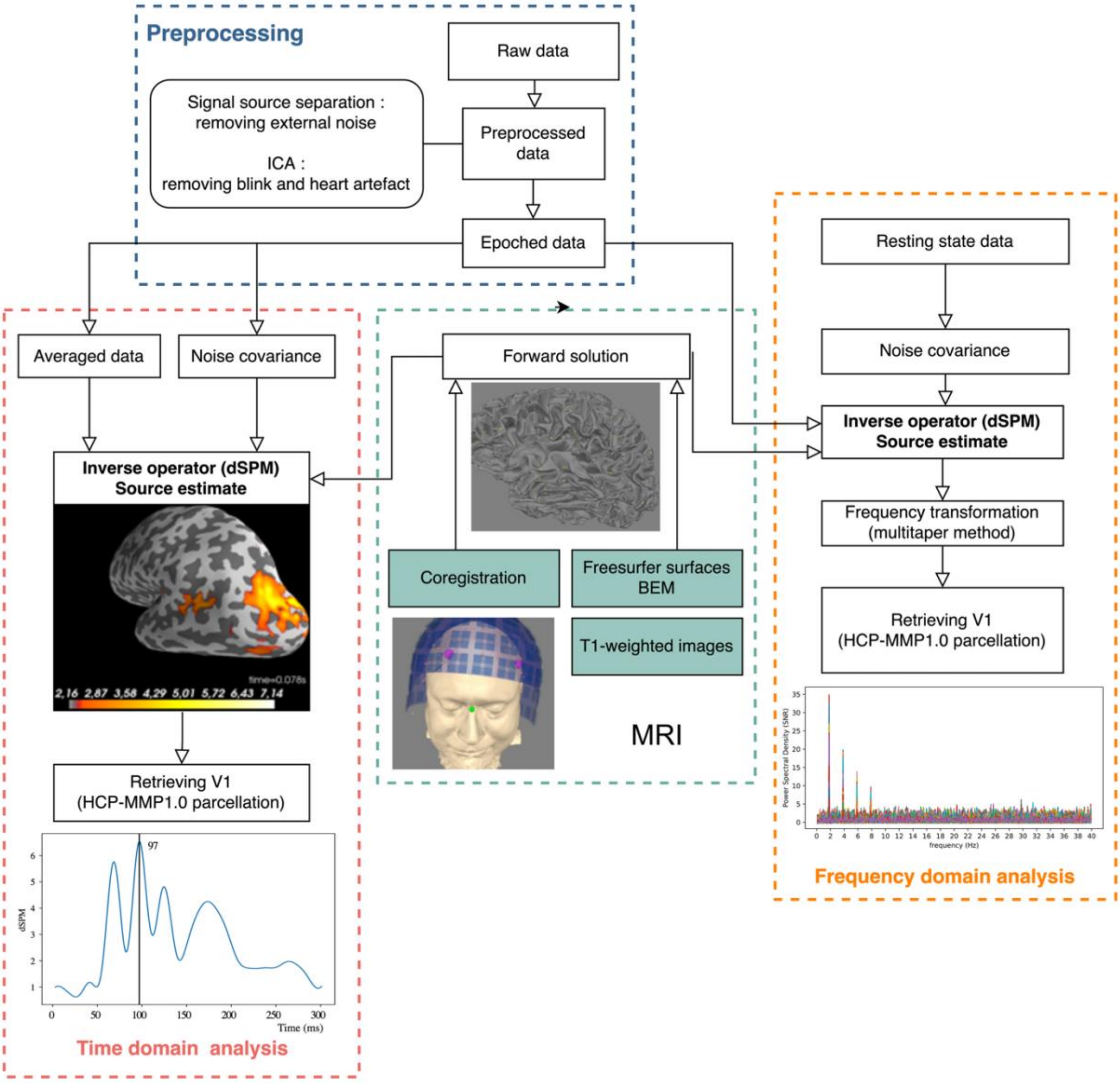

Supplementary Figure 1. MEG processing steps in temporal and frequency domains.

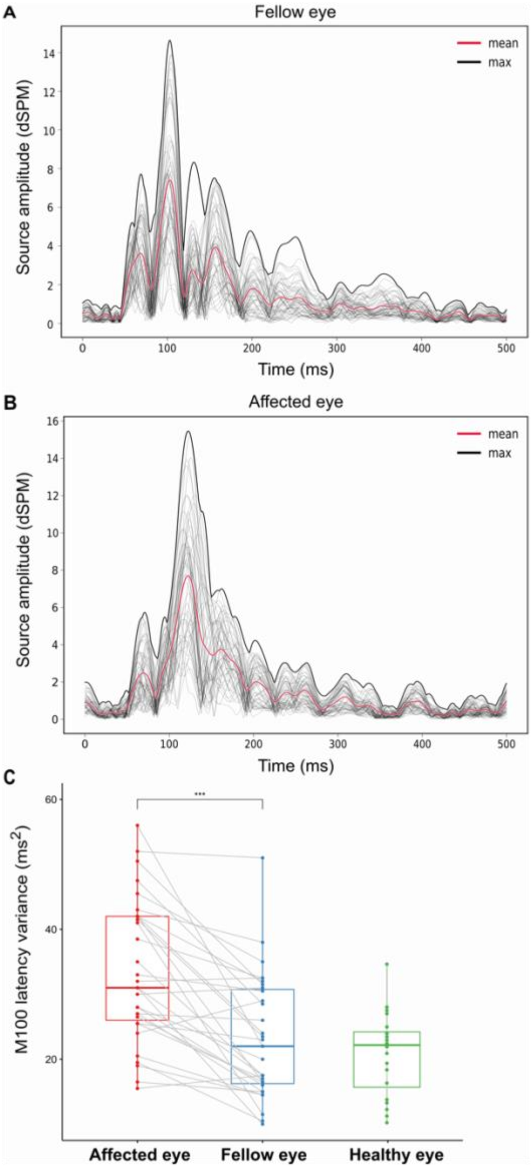

Supplementary Figure 2. M100 latency variance across V1 sources. Visual evoked field (VEF) for all the V1 sources was obtained after stimulation **(A)** of a fellow eye and (**B)** of an affected eye. (**C)** Boxplot of M100 latency variance calculated for all V1 sources in affected, fellow and healthy eyes. *Each data point represents a measurement from each eye for each patient.* Both eyes are displayed for the healthy subject group.*** *P* <0.001 in paired t-test

Supplementary Table 1. VEP P100 latency reproducibility between runs and between raters

	Rater	Eye	ICC (CI95%)	Absolute difference (ms)	TRV (ms)
Inter-run reproducibility	1	FE	0.75 (0.61-0.84)	3.8 ± 5.9	8.1
		AE	0.61 (0.37-0.78)	9.7 ± 15.4	21.5
	2	FE	0.71 (0.55-0.82)	4.1 ± 6.5	8.9
		AE	0.64 (0.41-0.80)	8.5 ± 14.2	20.4
Inter-rater reproducibility		FE	0.98 (0.97-0.99)	0.2 ± 2.2	3.1
		AE	0.99 (0.98-0.99)	0.8 ± 3.9	5.4

Fellow eye: $n = 29$ (one chart excluded for poor quality, two patients with only one run acquired)
Affected eye: $n = 21$ (due to undetectable responses in at least one of acquired runs).
VEP = Visual Evoked Potential, FE = Fellow Eye, AE = Affected Eye, ICC = Intraclass Correlation Coefficient, TRV = Test-Retest Variability
