# Supplementary material for: Magnetoencephalography biomarkers for assessing myelin content and neuronal function in acute optic neuritis
Source: Brain Commun. 2026 Jun 10;8(3):fcag218. doi: 10.1093/braincomms/fcag218 (PMC13289812; doi:10.1093/braincomms/fcag218)
Supplement: fcag218_Supplementary_Data [file fcag218_supplementary_data.zip › Supplementary Video Legends.docx]

**Supplementary Video 1.** Animation displaying source estimates over time across the brain’s cortical surface, following stimulation of a fellow eye (lateral view). *AU: arbitrary unit*

**Supplementary Video 2.** Animation displaying source estimates over time across the brain’s cortical surface, following stimulation of a fellow eye (medial view). *AU: arbitrary unit*
